# Survival outcomes of esophageal cancer patients with recurrence after curative treatments

**DOI:** 10.1186/s12885-023-11568-w

**Published:** 2023-11-01

**Authors:** Kotaro Sugawara, Daiji Oka, Hiroki Hara, Takako Yoshii, Hiroki Ushijima, Shigehiro Kudo, Takashi Fukuda

**Affiliations:** 1 Department of Gastroenterological Surgery, Saitama Cancer Center Hospital, 780 Komuro Inamachi, Kitaadachi-gun, Saitama, 362-0806 Japan; 2 Department of Gastroenterology, Saitama Cancer Center, Saitama, Japan; 3 Department of Radiation Oncology, Saitama Cancer Center, Saitama, Japan

**Keywords:** Esophageal carcinoma, Recurrence, Oligo Metastasis, GPS

## Abstract

**Background:**

Little is known about predictive factors for survival outcomes of esophageal carcinoma (EC) patients who developed recurrence after undergoing multimodal therapies. We aimed to investigate long-term outcomes and identify prognostic factors in patients with relapsed EC, focusing especially on those with oligometastasis (OM).

**Methods:**

EC patients who developed recurrence after curative treatments (radical esophagectomy or definitive chemoradiotherapy (dCRT)) between 2010 and 2017 were reviewed. Multivariate Cox hazards models were applied to determine independent predictors of poor post-recurrence survival (PRS).

**Results:**

In total, 178 patients were included. The median PRS was 12.9 months. Of the 178 patients, 98 had OM and 80 non-OM (NOM) disease. The survival outcomes of patients with OM were significantly better than those of patients with NOM (*P* < 0.01). Surgical treatments provided significantly better survival outcomes than CRT or chemo-/radiotherapy alone (3-year overall survival (OS); 78.1% vs. 42.5% vs. 28.9%, *P* < 0.01), mainly due to prolonging survival after the recurrence (3-year PRS 62.9% vs. 16.7% vs. 16.2%, *P* < 0.01). Multivariable analysis focusing on patients with OM revealed cStage III-IV disease (*P* < 0.01), high GPS at the time of recurrence (*P* = 0.02) and non-curative treatments (*P* < 0.01), to be independently associated with poor PRS. In contrast, in patients with NOM, no independent predictors for poor PRS were identified.

**Conclusions:**

The survival outcomes of patients with relapsed EC remain poor. Surgical treatments could provide survival benefits for patients with recurrent EC, especially for patients with OM.

**Supplementary Information:**

The online version contains supplementary material available at 10.1186/s12885-023-11568-w.

## Introduction

Esophageal cancer (EC) remains one of the refractory gastrointestinal malignancies [1, 2]. Recurrent disease is frequently observed even when curative treatments have achieved their objective [3]. Due to limited treatment options for disease relapses, the prognosis of patients who develop recurrence remains extremely poor [4, 5]. As part of the strategy for improving the survival outcomes of patients with recurrent EC, investigators have suggested several factors, such as time to recurrence, recurrence pattern and administering curative treatment for the recurrences, which might be useful for predicting post-recurrence survival [5–9].

Notably, patients with oligo metastasis (OM), a disease concept defined as a limited number of systemic metastatic tumors, reportedly have better survival outcomes than those with non-OM (NOM) disease [7, 10, 11]. In particular, a curative resection can provide a survival benefit for patients with OM, facilitating the provision of aggressive treatments for appropriately selected patients with OM [10, 12, 13].

Predictive factors for post-recurrence survival have been studied mainly in patients who developed OM after esophagectomy [8, 14]. Only a few studies have investigated factors predicting post-recurrence survival in patients undergoing multimodal therapies [7], although multimodal treatments including surgery and definitive chemoradiotherapy (dCRT) are currently applied for EC patients at all stages [1, 2].

Herein, we studied survival outcomes of patients who developed recurrence after receiving various multimodal therapies including surgery and dCRT. Furthermore, we investigated long-term outcomes and identified prognostic factors in patients with OM in comparison to those of patients with NOM.

## Patients and methods

### Patients

From January 2010 to December 2017, 711 consecutive patients in total with pathologically confirmed EC were treated at the Saitama Cancer Center. Initial treatment included upfront surgery (n = 173), neoadjuvant chemo(radio)therapy (n = 229) and dCRT (n = 309). Of these 711 patients, those who developed recurrence after curative (R0) esophagectomy or a clinical complete response to dCRT were included in the study. The clinical records of these patients were retrospectively reviewed from a prospectively maintained database. At the time of the final follow-up (January 2023), the median follow-up period was 61.6 months for the survivors. This retrospective study was approved by the local ethics committee of Saitama Cancer Center (ID: 1604).

### Studied criteria

Demographic data were collected prior to all treatments. Clinical and histological tumor staging was based on the TNM classification (UICC, 8th edition) [15]. As a nutritional parameter, Onodera’s prognostic nutritional index (PNI: 10 × albumin + 0.005 × total lymphocyte count) was calculated [16]. The Glasgow prognostic score (GPS) was determined and the neutrophil-to-lymphocyte ratio (NLR) was estimated as an inflammatory marker, as previously described [17, 18].

### Treatment strategy

Prior to treatment, the clinical stage was determined by each Multidisciplinary Tumor Board in accordance with the findings of esophagogastroduodenoscopy, computed tomography, endoscopic ultrasonography, and/or positron emission tomography. Patients were treated according to the Japan Esophageal Society guideline [19]. For patients with T1N1-3 or T2-4a (any N) disease, neoadjuvant chemotherapy followed by surgery was generally performed. For preoperative treatment, cisplatin (CDDP) plus 5-fluorouracil (5-FU) (CF) was administered as the standard therapy [20] and a regimen consisting of three drugs (CDDP, 5-FU, and docetaxel; DCF therapy) was optional [21]. As chemoradiotherapy, the CF regimen was added to radiation. For patients clinically diagnosed with cT4b disease and/or unresectable lymph node metastasis, dCRT was indicated as an initial treatment. dCRT was also applied for those who preferred nonsurgical treatment, regardless of the tumor stage. The patients who failed dCRT were candidates for salvage esophagectomy (SALV), if curative resection was deemed feasible.

### Surgical treatment

Our standard procedures consisted of subtotal esophagectomy along with en bloc lymph node dissection using a cervico-thoraco-abdominal approach. The operative thoracic approach was by video-assisted thoracoscopic surgery (VATS) or thoracotomy. We usually employed 3-field lymphadenectomy for the upper- and middle-thoracic EC and 2-field lymphadenectomy for lower-thoracic and abdominal EC. Basically, SALV was performed employing the McKeown procedure via right thoracoscopy with limited lymph node dissection, i.e., harvesting only lymph nodes that were swollen or suspected of harboring a recurrence [22]. A gastric conduit was passed through the retrosternal or posterior mediastinal route, and esophagogastric anastomosis was usually performed at the neck. The transmediastinal esophagectomy (abdominal-cervical approach) was selected for high-risk patients.

### Definitive chemoradiotherapy

dCRT is generally comprised of chemotherapy combined with 50.4 Gy of radiation or more to the main tumor and any metastases as well as more than 40 Gy of prophylactic radiation to the regional lymph nodes. As the first choice for the dCRT regimen, the standard-dose CF protocol of JCOG0303 was selected [23]. Additional CF therapy was continued as necessary following dCRT.

### Post-treatment follow-up evaluation

Postoperative surveillance was performed based on the Guidelines for Diagnosis and Treatment of Carcinoma of the Esophagus [19]. All patients were routinely followed up at 3-month intervals for the first 2 years, at 6-month intervals for the subsequent 3 years, and annually thereafter. Posttreatment surveillance for cancer recurrence included measuring blood tumor markers (SCC, CYFRA, P53 and CEA) and obtaining CT scans every 3–6 months after the patients had been discharged, and esophagogastroduodenoscopy was performed annually. Positron emission tomography scanning was added for patients in whom recurrence was indicated by other diagnostic modalities.

### Definitions of recurrence and oligo metastasis

Disease recurrences were classified as solely locoregional, solely distant, or combined [3]. Locoregional recurrences (LRRs) were located at the site of the primary tumor or in the locoregional lymph nodes, while distant recurrences were systemic or in nonregional lymph nodes, and combined recurrences were defined as the coexistence of locoregional and distant recurrences, regardless of the order of occurrence. Patients with recurrence at an anastomosis site or a regional lymph node in a cervical (including supraclavicular), mediastinal, or abdominal area were allocated to the LRR group. Patients with recurrence at a distant organ, such as the lung, brain, or liver, were allocated to the distant recurrence group; patients with recurrence at a lymph node surrounding the paraaortic regions were also included in this group.

In this study, OM was defined as fewer than five recurrences in one region, as previously reported [7, 10, 11].

### Treatment for the recurrence

In all of the recurrent cases, we decided on the management of therapy at a multidisciplinary conference. Treatment intents for disease recurrence were divided into surgery, chemo(radio)therapy, radiotherapy, or best supportive care (BSC). We performed metastasectomy basically when R0 resection was deemed possible. We resected only recurrent lesions. Prophylactic resection, such as radical neck dissection or lung lobectomy, was not performed. After metastasectomy, we discussed whether to perform adjuvant chemotherapy or prophylactic radiotherapy in Multidisciplinary Tumor Board [13].

CRT was mainly comprised of 5-FU and CDDP (CF) combined with a radiation dose of 50–60 Gy. We did not perform type-1 re-irradiation during the study period considering the toxicity from the cumulative doses [24]. For patients unable to undergo local treatment, such as surgery or CRT/RT, due to extensive recurrence sites, chemotherapy (CTx) comprised mainly of CF and/or taxanes was administered. For patients whose general conditions were poor, or who did not want to receive treatment for recurrences, BSC was selected.

### Statistical analysis

Categorical variables were expressed in numerical figures and percentages and compared using Fisher’s exact test or the χ2 test, as appropriate. Continuous variables were expressed as the median values (range) and compared using Wilcoxon’s rank-sum test (A Mann-Whitney U test). Overall survival (OS) was calculated from the start of the initial treatment. The disease-free interval (DFI) was defined as the interval between initial therapy for the primary lesion and the date of identification of recurrence. Post-recurrence survival (PRS) was calculated from the date of recurrence detection until death from any cause. Survival curves were constructed employing the Kaplan-Meier method, and the log-rank test was used to determine statistical significance, as appropriate.

To identify factors associated with survival, univariable and multivariable logistic regression analyses were performed. On the basis of literature and baseline characteristics, age, sex, histology, clinical stage, initial treatments, laboratory data at recurrence, recurrence pattern and treatment for the recurrence were included. Any variables that were significant (with a *P* value < 0.05) in the univariate analyses were included in multivariate analyses. Cox logistic regression was used to perform the multivariate analyses. Statistical analyses were carried out using JMP 16.0.0 (SAS Institute, Cary, NC).

## Results

### Characteristics of patients with recurrence after curative treatment

**Fig. 1:**
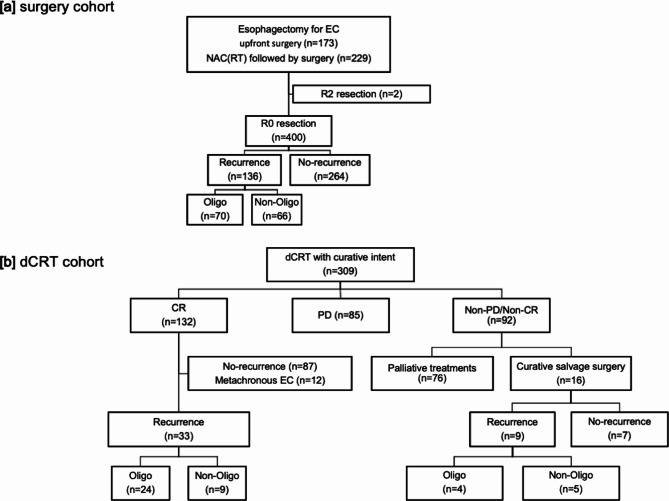
Study cohort. (**a**) Among 400 patients who underwent curative esophagectomy, 136 developed recurrences postoperatively. Of these, 70 had OM and 66 NOM disease. (**b**) In the dCRT group, CR was achieved in 132 patients and curative salvage surgery was performed in 16 patients. Among these 148 patients, 42 developed recurrences (28 OM, and 14 NOM)

Figure 1 presents the study cohort. Among 400 patients who underwent curative (R0) esophagectomy, 136 developed recurrent diseases postoperatively. Of these, 70 had OM disease and 66 NOM disease (Fig. 1a). Among 132 patients who achieved CR after dCRT, 33 developed recurrences (24 OM, and 9 NOM) (Fig. 1b). Curative (R0) SALV was performed in 16 patients. Of the 16 patients, 9 developed recurrences (4 OM, and 5 NOM) (Fig. 1b).

dCRT was generally selected for patients with upper thoracic EC and cStage III-IV disease (Supplementary Table 1). The incidence of LRR was significantly higher and surgery was more frequently performed in the dCRT group than in the surgery group (Supplementary Table 1).

Clinicopathological characteristics of 178 EC patients with recurrence are summarized in Table 1. The median DFI and PRS were 10.8 months (range; 1.2–113.8) and 12.9 months (range; 0.1–112.5), respectively. Clinical stage, initial treatments and DFI did not differ significantly between patients with OM and those with NOM. Patients with OM had lower levels of GPS and NLR, and higher PNI than those with NOM at the time of recurrence. Surgery was more frequently performed for OM than for NOM diseases (28.6% vs. 5.0%, *P* < 0.01).

**Table 1:** Characteristics of 178 patients with recurrence according to metastatic pattern

Variables	All	Non-oligo metastasis	Oligo metastasis	*P* value
	(n = 178)	(n = 80)	(n = 98)	
Age, y Median (range)	69 (39–84)	69 (39–82)	70 (45–84)	0.18
Sex, male/female	154 (86.5)/ 24 (13.5)	68 (85.0)/ 12 (15.0)	86 (87.8)/ 12 (12.2)	0.59
Tissue Type, SCC	145 (81.5)	63 (78.8)	82 (83.7)	0.41
Location				0.75
Lt-Ae	77 (43.3)	37 (46.3)	40 (40.8)	
Mt	67 (37.6)	28 (35.0)	39 (29.8)	
Ut-Ce	34 (19.1)	15 (18.7)	19 (19.4)	
cStage				0.36
I/II	19 (10.7)/ 39 (21.9)	5 (6.3)/ 19 (23.8)/	14 (14.3)/ 20 (20.4)	
III/IV	112 (62.9)/ 8 (4.5)	52 (65.0)/ 4 (5.0)	60 (61.2)/ 4 (4.1)	
Initial treatment				0.22
Primary surgery	53 (29.8)	26 (32.5)	27 (27.6)	
NAC(RT)	78 (46.6)	40 (50.0)	43 (43.9)	
dCRT	42 (23.6)	14 (17.5)	28 (28.6)	
Lymph node dissection				0.36
2-field	80 (44.9)	39 (48.8)	41 (41.8)	
3-field	98 (55.1)	41 (51.2)	57 (58.2)	
DFI, months Median (range)	10.8 (1.2-113.8)	8.9 (1.2–76)	11.9 (1.4-113.8)	0.11
Recurrence pattern				< 0.01
LRR	75 (42.1)	27 (33.8)	48 (48.9)	
Distant	75 (41.0)	25 (31.3)	50 (51.1)	
Combined	28 (16.9)	28 (35.0)	0 (-)	
Data at the time of recurrence				
GPS 1–2	40 (22.9)	24 (30.0)	16 (16.5)	0.03
PNI, Median (range)	45.6 (27.8–61)	45.1 (27.8–61)	46.4 (29.2–57.9)	0.09
NLR, Median (range)	2.6 (0.5–28.9)	2.7 (0.8–28.9)	2.5 (0.5–12.8)	0.04
Elevated tumor marker	111 (62.4)	53 (66.3)	58 (59.2)	0.33
Treatment for the recurrence				< 0.01
Surgery	32 (18.0)	4 (5.0)	28 (28.6)	
CRT	25 (14.0)	15 (18.8)	10 (10.2)	
CTx or RT	94 (52.8)	45 (56.3)	49 (50.0)	
BSC	27 (15.2)	16 (20.0)	11 (11.2)	
PRS, months Median (range)	12.9 (0.1-102.5)	9 (0.1-100.7)	16.2 (0.1-102.5)	< 0.01

Abbreviations: SCC, squamous cell carcinoma; NAC, neoadjuvant chemotherapy; NACRT, neoadjuvant chemoradiotherapy; dCRT, definitive chemoradiotherapy; DFI, disease-free interval; LRR, locoregional recurrence; GPS, Glasgow prognostic score; PNI, prognostic nutritional index; NLR, neutrophil lymphocyte ratio; CTx, chemotherapy; BSC, best supportive care; PRS, post-recurrence survival

### Survival outcomes

**Fig. 2:**
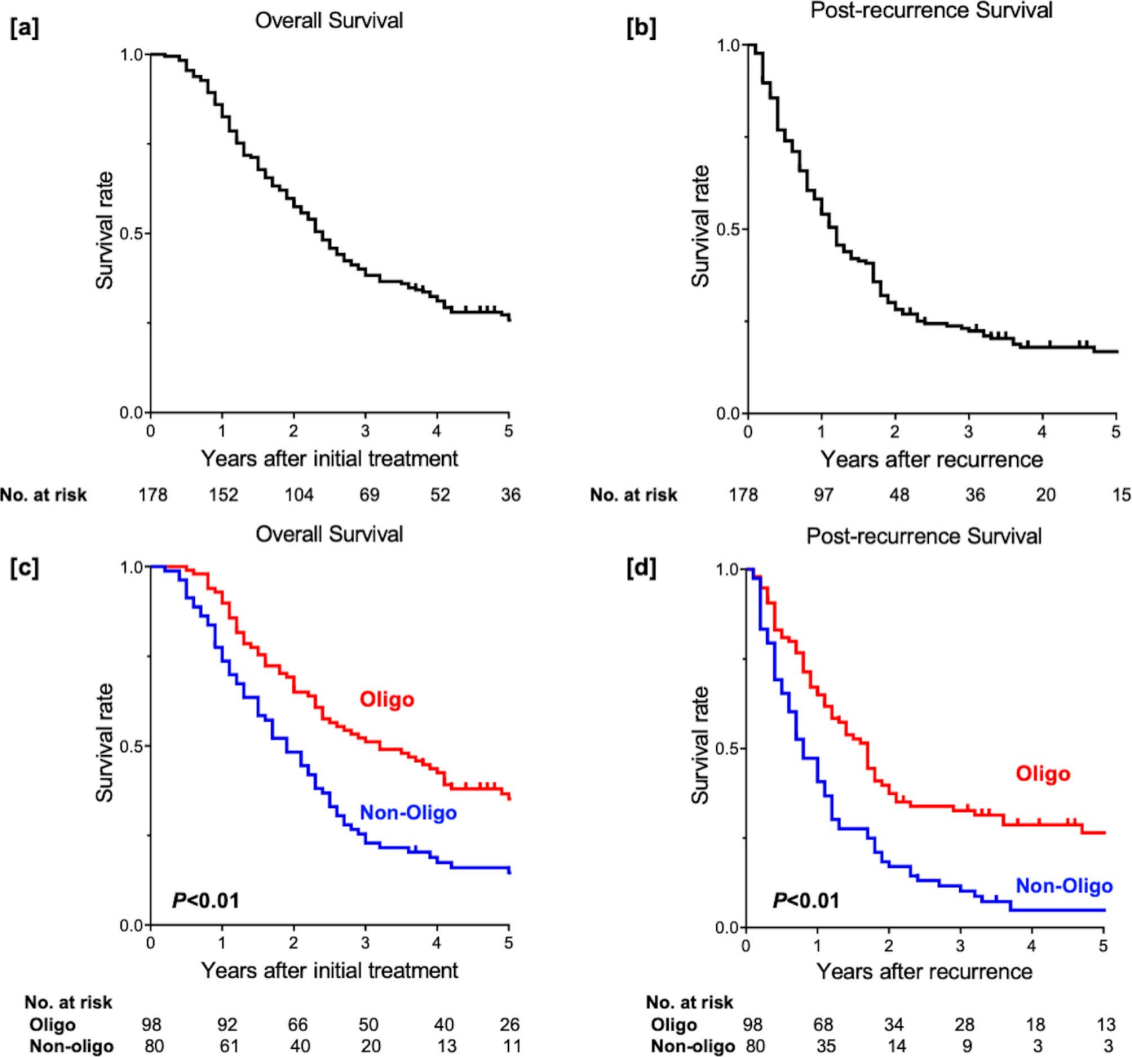
Survival outcomes. The (**a**) OS and (**b**) PRS curves of all patients. Patients with OM showed significantly better (**c**) OS and (**d**) PRS than those with NOM (both *P* < 0.01)

The 3- and 5-year OS of EC patients with recurrence were 39.5% and 26.5%, respectively (Fig. 2a). The 1- and 3-year PRS of EC patients with recurrence were 55.4% and 22.6%, respectively (Fig. 2b). The survival outcomes of patients with OM were significantly better than those of patients with NOM (3-year OS; 52.2% vs. 24.1%, 1-year PRS 43.5% vs. 65.4%, both *P* < 0.01, Fig. 2c, d).

**Fig. 3:**
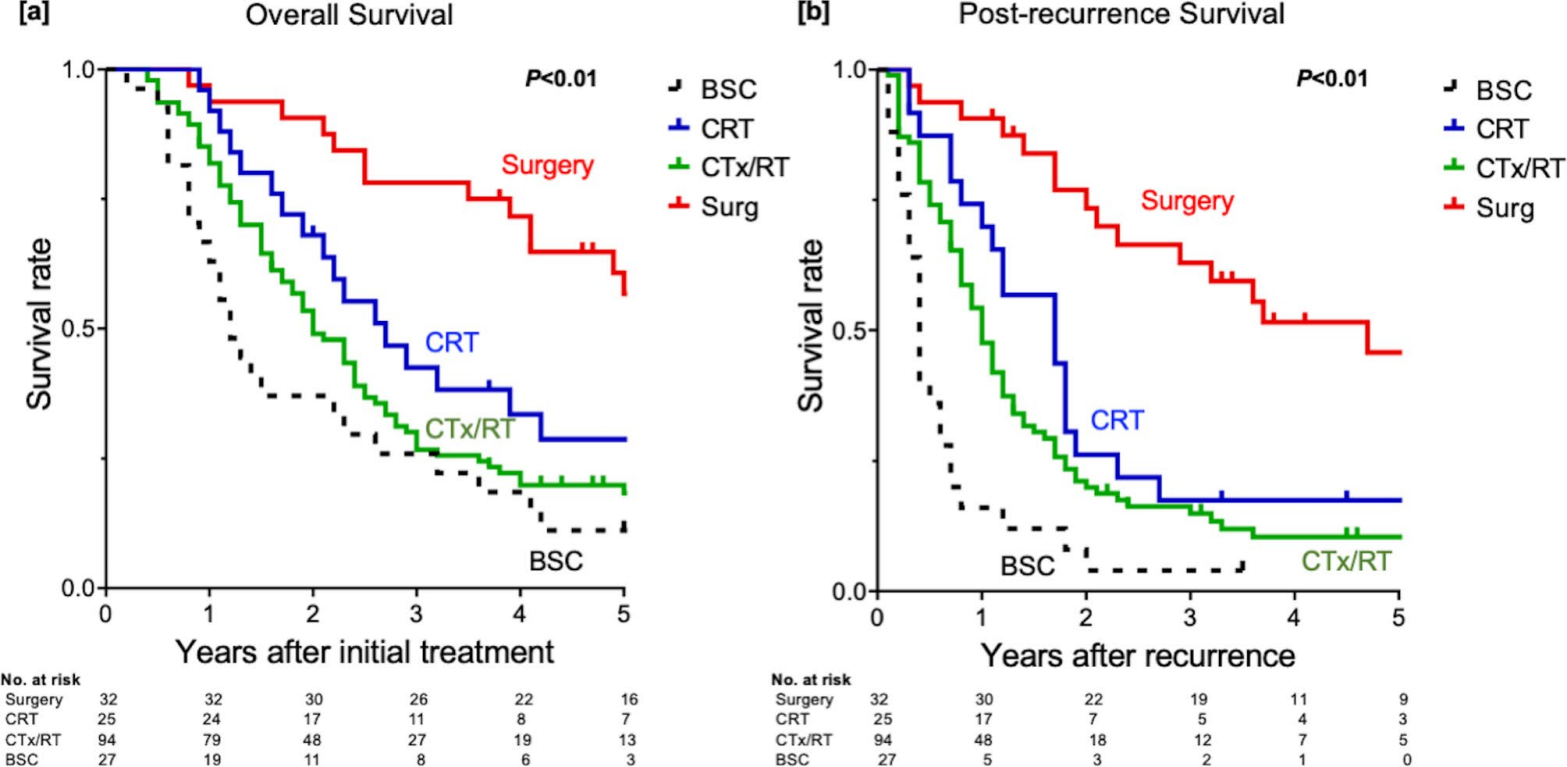
Survival outcomes according to treatments for the recurrence. (**a**) Surgical treatments provided significantly better survival outcomes than CRT or CTx/RT (*P* < 0.01), (**b**) mainly due to prolonging survival after the recurrence (*P* < 0.01)

Treatments for disease recurrence included surgery (n = 32), CRT (n = 25), CTx alone (n = 71), RT alone (n = 23) and BSC (n = 27). Among 32 patients who underwent surgery for the recurrence, 5 (3 with locoregional LN recurrences, 1 with cervical LN recurrence and 1 with brain metastasis) were microscopically diagnosed as having positive circumferential resection margin (R1 resection). Surgical treatments provided significantly better survival outcomes than CRT or CTx/RT (3-year OS; 78.1% vs. 42.5% vs. 28.9%, *P* < 0.01, Fig. 3a), mainly due to prolonging survival after the recurrence (3-year PRS 62.9% vs. 16.7% vs. 16.2%, *P* < 0.01, Fig. 3b, Supplementary Fig. 1).

Overall, post-recurrence survival is the major determinant of the long-term outcomes of EC patients with recurrence.

### Factors predicting poor post-recurrence survival

Next, we endeavored to identify clinical factors potentially useful for predicting post-recurrence survival. Univariable analysis and subsequent application of the multivariable Cox proportional hazards model revealed shorter DFI (< 1 year) (HR 1.57, 95%CI 1.07–2.31, *P* = 0.02), GPS 1 or 2 (HR 1.81, 95%CI 1.08–3.03, *P* = 0.02) and non-surgical treatments (*P* < 0.01), to be independently associated with poor PRS outcomes (Table 2). Further, we studied differences in factors for predicting post-recurrence survival between patients in the dCRT cohort and those in the surgery cohort (Supplementary Table 2). Non-surgical treatments consistently yielded a negative impact on PRS outcomes regardless of the initial treatments. Shorter DFI and high GPS was an independent predictor for poor PRS only in the surgery cohort and in the dCRT cohort, respectively.

**Table 2:** Cox hazards model for survival after recurrence (n = 178)

Variables	Univariable analysis			Multivariable analysis		
	HR	95% CI	*P* value	HR	95% CI	*P* value
Age > 65	0.96	0.64–1.29	0.59			
Male	1.19	0.73–1.97	0.47			
Histology, SCC	0.92	0.59–1.40	0.69			
cStage III-IV (vs. I-II)	1.41	0.98–2.04	0.06			
dCRT	0.76	0.51–1.13	0.16			
DFI < 1 year	1.82	1.29–2.55	< 0.01	1.57	1.07–2.31	0.02
Data at recurrence						
GPS 1,2 (vs. GPS 0)	2.86	1.94–4.21	< 0.01	1.81	1.08–3.03	0.02
PNI < 45	1.62	1.16–2.28	< 0.01	1.07	0.71–1.61	0.76
NLR high (vs. low)	1.29	0.92–1.79	0.14			
elevated TM	1.39	0.99–1.98	0.06			
Non-Oligo (vs. oligo)	2.09	1.50–2.93	< 0.01	1.37	0.96–1.96	0.08
Treatment for the recurrence						
Surgery	Ref			Ref		
CRT/CTx/RT	3.44	2.09–5.67	< 0.01	3.19	1.84–5.54	< 0.01
BSC	8.65	4.76–15.75	< 0.01	6.74	3.43–13.25	< 0.01

Abbreviations: SCC, squamous cell carcinoma; dCRT, definitive chemoradiotherapy; GPS, Glasgow prognostic score; PNI, prognostic nutritional index; NLR, neutrophil lymphocyte ratio; TM, tumor marker; CTx, chemotherapy; BSC, best supportive care

### Treatment details and prognostic factors in patients with OM

**Fig. 4:**
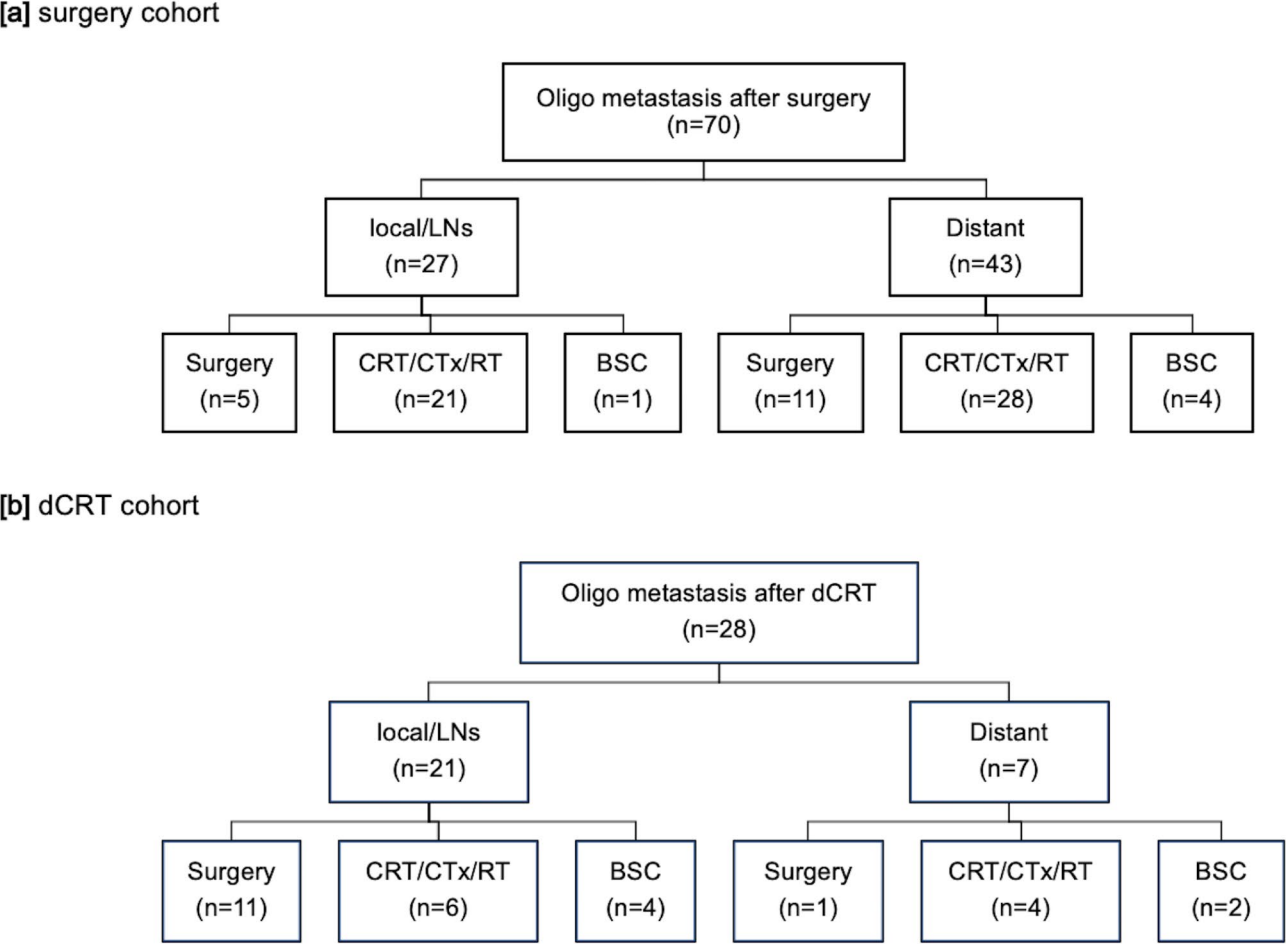
Details of treatments for oligometastasis

Last, we investigated prognostic factors focusing on patients with OM. The details of recurrence site and treatment modalities in these patients are shown in Fig. 4. The incidence of oligo LRR was significantly higher in the dCRT group than in the surgery group (75.0% vs. 38.6%, *P* < 0.01). Nearly half of the patients with oligo LRR received surgery in the dCRT group (11/21, 52.4%), while curative resection was feasible for only a quarter of the patients with oligo LRR in the surgery group (5/27, 18.5%). A quarter of patients (11/43) with distant OM after curative surgery were treated surgically, whereas curative resection was not achievable in most of the patients with distant OM after dCRT (Fig. 4).

Multivariable analysis revealed cStage III-IV disease (HR 2.23, 95%CI 1.26–3.96, *P* < 0.01), GPS 1 or 2 (HR 2.44, 95%CI 1.12–5.31, *P* = 0.02) and non-surgical treatments (*P* < 0.01), to be independently associated with poor PRS outcomes in patients with OM (Table 3). In patients with NOM, only BSC was an independent predictive factor for poor PRS outcomes (Table 3).

**Table 3:** Differences in prognostic factors between patients with OM and NOM

Variables	Univariable analysis			Multivariable analysis		
	HR	95% CI	*P* value	HR	95% CI	*P* value
**Patients with OM**						
Age > 65	0.68	0.42–1.12	0.13			
Histology, SCC	1.27	0.63–2.57	0.51			
cStage III-IV (vs. I-II)	1.89	1.09–3.24	0.02	2.23	1.26–3.96	< 0.01
dCRT	0.81	0.47–1.38	0.43			
GPS 1,2 (vs. GPS 0)	3.07	1.68–5.62	< 0.01	2.44	1.12–5.31	0.02
PNI < 45	1.69	1.03–2.75	0.04	1.11	0.59–2.04	0.75
DFI < 1 year	1.93	1.17–3.16	< 0.01	1.32	0.77–2.26	0.31
Treatment for the recurrence						
Surgery	Ref					
CRT/CTx/RT	3.78	1.94–7.39	< 0.01	4.62	2.32–9.19	< 0.01
BSC	10.92	4.58–26.03	< 0.01	11.29	4.57–27.94	< 0.01
**Patients with NOM**						
Age > 65	1.11	0.67–1.85	0.69			
Histology, SCC	0.75	0.43–1.29	0.29			
cStage III-IV (vs. I-II)	0.88	0.54–1.45	0.63			
dCRT	0.85	0.47–1.53	0.59			
GPS 1,2 (vs. GPS 0)	2.18	1.29–3.68	< 0.01	1.74	0.94–3.21	0.08
PNI < 45	1.43	0.89–2.28	0.13			
DFI < 1 year	1.72	1.08–2.76	0.03	1.38	0.79–2.41	0.25
Treatment for the recurrence						
Surgery	Ref					
CRT/CTx/RT	1.51	0.54–4.19	0.43	1.71	0.62–4.76	0.31
BSC	3.34	1.09–10.23	0.03	3.23	1.04–9.97	0.04

Abbreviations: SCC, squamous cell carcinoma; dCRT, definitive chemoradiotherapy; GPS, Glasgow prognostic score; PNI, prognostic nutritional index; NLR, neutrophil lymphocyte ratio; TM, tumor marker; CTx, chemotherapy; BSC, best supportive care

## Discussion

The present study demonstrates the aggressive nature of EC with high recurrence rates after curative treatments [3]. In our series, approximately 30% of patients developed recurrent disease after curative treatments, 80% of all recurrences were detected within 2 years, and median post-recurrence survival was 12.9 months, observations which are in line with previous studies [4, 5, 8]. We also revealed survival outcomes of EC patients with recurrence to be mainly dependent on the post-recurrence survival; therefore, optimizing the treatment strategy for recurrence is crucial for improving the survival outcomes of EC patients [7].

Recent studies have revealed isolated recurrence, i.e., OM, to be associated with favorable outcomes after recurrence in EC patients [7, 8, 10, 12, 14]. The favorable survival outcomes of patients with OM are mainly due to the high probability of achieving curative treatment for their disease relapses. In fact, our present study revealed surgical treatments to provide significantly better survival outcomes than CRT or CTx/RT, mainly due to prolonging survival after the recurrence.

In the present study, we investigated factors other than the presence of curative treatments which might be useful for predicting post-recurrence survival. Our results show short DFI (< 1 year) and high GPS at the time of recurrence, as well as non-surgical treatments, to be independently associated with poor post-recurrence survival. The survival impacts of short DFI [5, 8, 17], type of recurrence [5, 6, 9, 14, 17], the presence of curative treatments for the recurrence [5, 7] and NLR [17] have been proposed in patients undergoing surgery for EC. Our study suggested these factors to be useful for predicting post-recurrence survival not only in patients undergoing surgery but also in those receiving multimodal therapies including surgery and dCRT. Of note, OM was not independently associated with good survival outcomes in our series. The rate of surgical resection was significantly higher in the OM group than in the non-OM group, thereby possibly diminishing the independent survival impact of OM.

Inflammation-based prognostic scores are reportedly useful for predicting long-term outcomes for EC patients with various tumor stages [18, 25, 26] and with recurrence [17]. Prior studies revealed short DFI to be associated with poor survival outcomes in patients with recurrence after EC surgery [5, 8, 17]. These factors are easily estimated at the timing of recurrence, thereby making them useful for clinicians aiming to estimate the survival outcomes of their patients.

It is noteworthy that we first studied predictive factors for post-recurrence survival in patients with OM in comparison to those with NOM [7, 8, 14]. In patients with OM, several factors (cStage III-IV, high GPS and non-surgical treatments) were identified as independent predictors of poor PRS. In marked contrast, in patients with NOM, only BSC independently predicted poor PRS. Our findings highlight the difference in prognostic factors between patients with OM and those with NOM. The survival outcomes of those with NOM were quite poor regardless of patient status, original tumor background and treatments.

We identified several factors useful for predicting survival in patients with OM. The finding that cStage III-IV disease is independently associated with poor PRS is in line with a recent study [14], although the precise mechanism has yet to be clarified. Importantly, our observations, together with those from previous studies [7, 12, 13], suggest that aggressive surgical treatment should be considered when the recurrent lesion is solitary or localized. Of note, short DFI was not an independent predictor of poor PRS in patients with OM. This finding suggests that surgeons should not hesitate to resect oligometastases even when the disease develops relatively soon after the attempt to achieve curative treatment [7, 14].

Although early identification and aggressive treatment for oligometastatic recurrence might improve survival [7, 8, 10, 12], optimal treatment strategies for recurrent EC have yet to be determined. Importantly, the significance of resection reportedly differs among organs harboring recurrent disease. Pulmonary metastasectomy is reportedly efficacious for solitary pulmonary metastasis [27–29]. On the other hand, the benefit of resection for hepatic metastases remains controversial [28, 30]. Still, patients should be considered for resection on an individual basis with the input of a multidisciplinary team of specialists [30].

Limitations need to be considered when interpreting the results of this study. First, the largest bias of this study was that surgical indications and treatment modalities for the recurrent lesions were determined mainly by each clinician and patient. We tried to remove the selection bias as much as possible by conducting a multidisciplinary conference for all recurrent patients; however, the choice of treatment for recurrence remains arbitrary [7, 14]. A comparative study with a large cohort is needed to confirm our observations. Second, our cohort was comprised of patients receiving various treatment modalities. Recurrence patterns and timing differ according to the initial treatments [3, 4, 31], which might have affected the results. Third, it is not possible to discern whether the observed isolated solid organ disease represents true OM or the first clinically apparent presentation of widespread metastatic disease. Finally, this was a single-institution, retrospective study. We anticipate that a multi-institutional collaborative study with a large cohort would achieve a more convincing result.

In conclusion, the survival outcomes of patients with relapsed EC remain poor. Surgical treatments might provide survival benefits for patients with relapsed EC, especially for patients with OM, due to prolonging survival after the recurrence. Further studies are needed to determine optimal treatment strategies for the tumor entity.

### Electronic supplementary material

Below is the link to the electronic supplementary material.

**Supplementary Material 1: Supplementary Table 1**. Characteristics of 178 patients with recurrence according to the initial treatments

**Supplementary Material 2: Supplementary Figure 1**. DFI and PRS according to treatment modalities and recurrence patterns

## Data Availability

The datasets used and/or analyzed during the current study are available from the corresponding author upon reasonable request.

## Abbreviations

BSC: Best supportive care
CI: Confidence intervals
CTx: Chemotherapy
dCRT: Definitive chemoradiotherapy
DFI: Disease-free interval
EC: Esophageal carcinoma
GPS: Glasgow prognostic score
HR: Hazard ratios
LRR: Locoregional recurrence
NLR: Neutrophil-to-lymphocyte ratio
OM: Oligo metastasis
OS: Overall survival
PNI: Prognostic nutritional index
PRS: Post-recurrence survival
SALV: Salvage esophagectomy
VATS: Video-assisted transthoracic surgery
