# Supplementary material for: Survival outcomes of esophageal cancer patients with recurrence after curative treatments
Source: BMC Cancer. 2023 Nov 1;23:1051. doi: 10.1186/s12885-023-11568-w (PMC10619310; doi:10.1186/s12885-023-11568-w)
Supplement: Supplementary file 1 — Supplementary Material 1: Supplementary Table 1. Characteristics of 178 patients with recurrence according to the initial treatments [file 12885_2023_11568_MOESM1_ESM.docx]

| **Supplementary Table 1.**  **Characteristics of 178 patients with recurrence according to the initial treatments** | | | |
| --- | --- | --- | --- |
| Variables | dCRT  (n=42) | Surgery  (n=136) | *P* value |
| Age, y Median (range)  Sex, male/female  Tissue Type, SCC  Location, Lt-Ae/Mt/Ut-Ce  cStage  I/II  III/IV  DFI, months; Median (range)  Recurrence pattern  LRR/Distant/Combined  Oligo/Non-oligo  Data at the time of recurrence  GPS 1-2  PNI, Median (range)  NLR, Median (range)  Elevated tumor marker  Treatment for the recurrence  Surgery/CRT  CTx or RT/BSC  PRS, months Median (range) | 69 (39-82)  38 (90.5)/ 4 (9.5)  41 (97.6)  7 (16.7)/ 22 (52.4)/ 13 (30.9)  4 (9.5)/ 3 (7.1)  33 (78.6)/ 2 (4.8)  12.7 (5.8-113.8)  26 (61.9)/ 13 (31.0)/ 3 (7.1)  28 (66.7)/ 14 (33.3)  8 (19.5)  44.8 (29.2-57.1)  3.2 (1.1-8.9)  20 (47.6)  16 (38.1)/ 2 (4.8)  17 (40.5)/ 7 (16.7)  15.6 (0.1-102.5) | 70 (45-84)  116 (85.3)/ 20 (14.7)  104 (76.5)  70 (51.5)/ 45 (33.1)/ 21 (15.4)  15 (11.0)/ 36 (26.5)  79 (58.1)/ 6 (4.4)  8.9 (1.2-85.1)  49 (36.0)/ 62 (45.6)/ 25 (18.4)  70 (51.5)/ 66 (48.5)  32 (23.9)  46.3 (27.8-61)  2.5 (0.5-28.9)  91 (66.9)  16 (11.8)/ 23 (16.9)  77 (56.6)/ 20 (14.7)  12.5 (0.1-100.7) | 0.23  0.37  <0.01  <0.01  0.03  <0.01  <0.01  0.08  0.55  0.26  0.54  0.03  <0.01  0.09 |
| Abbreviations: SCC, squamous cell carcinoma; NAC, neoadjuvant chemotherapy; NACRT, neoadjuvant chemoradiotherapy; dCRT, definitive chemoradiotherapy; DFI, disease-free interval; LRR, locoregional recurrence; GPS, Glasgow prognostic score; PNI, prognostic nutritional index; NLR, neutrophil lymphocyte ratio; CTx, chemotherapy; BSC, best supportive care; PRS, post-recurrence survival | | | |

| **Supplementary Table 2. Predictors for survival after recurrence according to the initial treatments** | | | | | | |
| --- | --- | --- | --- | --- | --- | --- |
| Variables | Univariable analysis | | | Multivariable analysis | | |
|  | HR | 95% CI | *P* value | HR | 95% CI | *P* value |
| **Patients in the dCRT cohort**  Age >65  Histology, SCC  cStage III-IV (vs. I-II)  DFI < 1 year  labo data at recurrence  GPS 1,2 (vs. GPS 0)  PNI <45  Non-Oligo (vs. oligo)  Treatment for the recurrence  Surgery  CRT/CTx/RT  BSC  **Patients in the surgery cohort**  Age >65  Histology, SCC  cStage III-IV (vs. I-II)  NAC(RT)  DFI < 1 year  labo data at recurrence  GPS 1,2 (vs. GPS 0)  PNI <45  Non-Oligo (vs. oligo)  Treatment for the recurrence  Surgery  CRT/CTx/RT  BSC | 0.48  0.33  1.48  1.13  2.51  1.66  2.23  Ref  3.53  10.78  1.07  1.02  1.51  0.97  2.14  3.03  1.73  1.93  Ref  4.09  10.72 | 0.22-1.06  0.04-2.55  0.5204.24  0.56-2.30  1.06-5.99  0.81-3.39  1.10-4.52  1.53-8.16  3.61-32.21  0.72-1.58  0.65-1.59  1.01-2.24  0.68-1.46  1.45-3.18  1.95-4.71  1.17-2.55  1.32-2.83  1.78-9.42  4.23-27.19 | 0.07  0.29  0.47  0.73  0.03  0.16  0.02  <0.01  <0.01  0.75  0.92  0.04  0.97  <0.01  <0.01  <0.01  <0.01  <0.01  <0.01 | 4.11  1.69  Ref  4.35  10.46  1.72  1.59  1.18  1.25  Ref  3.19  6.71 | 1.37-12.29  0.77-3.71  1.62-11.71  3.25-33.64  1.09-2.71  0.85-2.98  0.71-1.96  0.83-1.86  1.34-7.58  2.47-18.22 | 0.01  0.19  <0.01  <0.01  0.02  0.14  0.51  0.28  <0.01  <0.01 |
| Abbreviations: CRT, definitive chemoradiotherapy; SCC, squamous cell carcinoma; d NAC(RT), neoadjuvant chemo(radio)therapy; GPS, Glasgow prognostic score; PNI, prognostic nutritional index; CTx, chemotherapy; BSC, best supportive care | | | | | | |
