# Supplementary material for: Survival outcomes of esophageal cancer patients with recurrence after curative treatments
Source: BMC Cancer. 2023 Nov 1;23:1051. doi: 10.1186/s12885-023-11568-w (PMC10619310; doi:10.1186/s12885-023-11568-w)
Supplement: Supplementary file 2 — Supplementary Material 2: Supplementary Figure 1. DFI and PRS according to treatment modalities and recurrence patterns [file 12885_2023_11568_MOESM2_ESM.docx]

**Supplementary Figure 1. DFI and PRS according to treatment modalities and recurrence patterns**

**
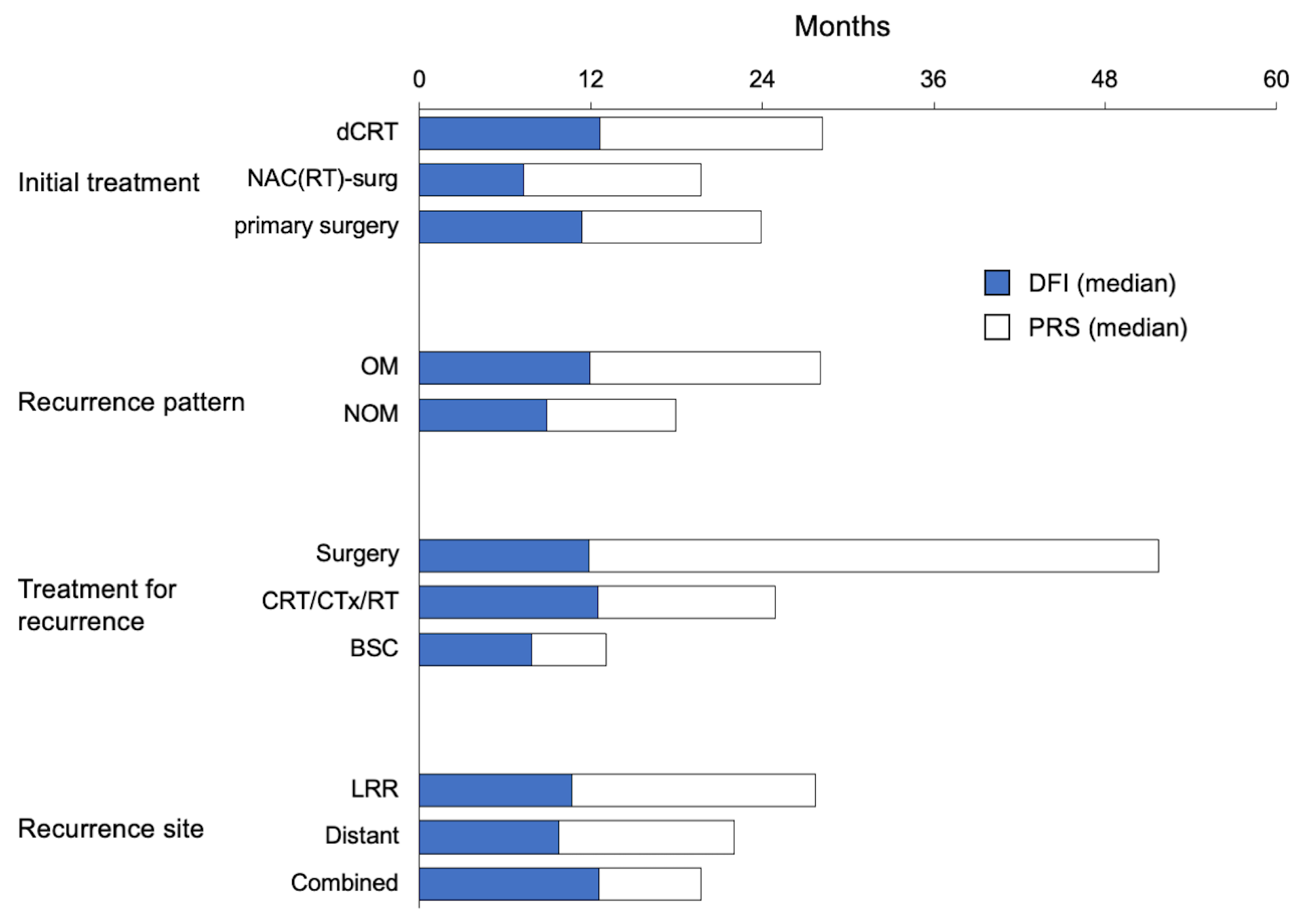
**

Surgical treatments provided significantly better survival outcomes than CRT or CTx/RT, mainly due to prolonging survival after the recurrence.
